# The Specific Treatment of Tuberculosis

**Published:** 1904-06

**Authors:** 


### THE SPECIFIC TREATMENT OF TUBERCULOSIS.*

During the past twenty years numerous attempts have been made to discover a specific against tuberculosis, but none of them have proven entirely successful. All of us can remember the excitement which the discovery of tuberculin, or "Koch's Lymph," as it was called, caused throughout the world, and the rush of patients as well as physicians to Berlin—the one in the hope of being cured, the other to learn the new method of treating the dreaded malady. Numerous modifications of the original tuberculin have been proposed by Koch himself and by others, but all of them have failed of their object—most of them in even greater degree than the first. It can not be denied that tuberculin has a certain power to fortify the body against the invasion of the tubercle bacillus, and with suitable precautions has a curative property in early cases of tuberculosis, both of these results being due to the production of a degree of immunity.

The artificial production of immunity against tuberculosis presents many difficulties, and nature has not given us a hint of procedure as she has done in those diseases in which one attack protects against another. Relapse is the common clinical history, though if must be acknowledged that healed tubercular lesions are frequently met with on the autopsy table in those who were not suspected of the disease during life. It would appear that in these cases we have a certain degree of acquired immunity as the result of the infection. There is good reason to believe that the results would be better and more evident were it not for the extreme frequency of mixed and secondary infections, which not only mar the clinical picture, but at the same time insure a fatal issue; and destroy any possibility of immunity being established.

The first attempts at a specific treatment of tuberculosis were based on the idea that certain bacteria were inimical to the growth of the tubercle bacillus, and these supposedly hostile organisms were injected into the tuberculous person. The best known, perhaps, of these methods was that of Petruschky, who proposed to cure lupus by the injection of streptococcus.

The next step was to attempt to produce a passive immunity in susceptible animals by the injection of the serum of animals supposed to be naturally immune to tuberculosis. The serum of dogs, goats and horses was used by different experimenters, and some apparently good results were reported. It has since been shown that these animals are not really immune to tuberculosis, and the good

*Read by Mazyck P. Ravenel, M.D., of Philadelphia, Bacteriologist of the State Live Stock Sanitary Board of Pennsylvania; Assistant Medical Director of the Henry Phipps Institute for the Study, Treatment and Prevention of Tuberculosis, at the meeting of the Medical Society of the State of Pennsylvania, held at York, September 22 to 24, 1903.

effect observed is readily explained by recent researches. Certainly there was nothing specific in it. It has been since shown by Maragliano that the serum of a large number of animals, including man, has a certain power against the tubercle bacillus.

After this the idea arose that the blood of tuberculous animals contains a substance inimical to the tubercle bacillus, and various experiments were tried with the serum of animals infected for the purpose. The treatment of man with such serum was tried, but fell into disuse.

At the present time all other methods have given way to the rational one of producing artificial immunity against tuberculosis in animals, and employing the anti-bodies thus brought into being for the treatment of man. While this is generally conceded to be the only practicable method, there is much difference of opinion among workers as to the details of procedure necessary to bring about the best result.

The brilliant results achieved in purely toxic diseases like diphtheria and tetanus naturally led experimenters to follow similar methods, but it was soon demonstrated that the conditions to be met were very different in their nature. In the purely toxic diseases immunity is brought about in animals by gradually increasing doses of the soluble toxins produced during the growth of the specific organism in the culture medium.

The toxins of the tubercle bacillus are of two kinds—those formed in the culture medium, and generally spoken of as extra-cellular; and those fixed in the protoplasm, and called intra-cellular. The latter are held very firmly by the tubercle bacillus, and are of great importance, since through their action even dead bacilli are able to produce typical tubercles in the tissues. In order, therefore, to produce a serum which will be efficacious against tuberculosis, it is necessary to immunize our animals against these poisons. Many attempts have been made along these lines. With the belief that Koch's old tuberculin contained all of the toxic products of the tubercle bacillus this substance was used in many of the early experiments. Animals acquire in this way a considerable tolerance for tuberculin, so that doses which would otherwise be fatal may be given, but the serum of such animals does not contain anti-toxin capable of neutralizing the minimum fatal dose of tuberculin, nor is there any certain immunity produced against inoculation with tubercle baccilli. It is said that with Koch's new tuberculin (T. R.) immunity against living and virulent tubercle bacilli can be produced, but this is not generally accepted.

Maragliano, by using the extra- and intra-cellular toxins combined has been more successful than any other worker along these lines, probably because he has succeeded more nearly than any other in obtaining all the toxins formed by the tubercle bacillus.

With the serum of immunized animals, Maragliano has succeeded in producing a marked degree of passive immunity in other animals, and also in man. Under its use he has seen the agglutinating power of the blood of a healthy man increase until a dilution of one part in fifty was efficient. In animals he has not only seen a like elevation in agglutinating power, but has enabled them to resist inoculation into the circulation with tubercle bacilli.

Maragliano's serum has been more largely employed clinically than any other, upwards of three thousand cases having been treated with it since 1895, apparently with good results. Outside of Italy, however, it is but little known, and the medical profession generally has not given its endorsement to it. Maragliano himself does not claim complete success, but we must accord to him the credit for having made most laborious and painstaking researches which have greatly advanced our knowledge of the subject.

Besides the immunity against toxines already mentioned, we have come to recognize another type, or bacteriolytic immunity, which is much more complex, and much less understood. It is usually produced by inoculation with living germs which have been attenuated in virulence by the action of heat or antiseptics, or have lost their virulence by prolonged cultivation on artificial culture media. In the case of the tubercle bacillus we have somewhat the same effect brought about by the separation of the organism into races by prolonged habitation in different species of animals, thus acquiring an increased virulence for the species in which it dwells, but losing pathogenic power for some of the other species. The great exception to this rule is the bovine tubercle bacillus, which has greatly increased pathogenic power for practically all experimental animals. We have at least three races of tubercle bacillus—human, bovine and avian—and it has been shown that the weaker of these races have a vaccinal power against the more virulent, a powerful argument in favor of the genetic unity of all tubercle bacilli.

By proceeding along the lines just indicated very brilliant results have been achieved in the last few years. As early as 1889 Daremberg said: "When one injects very small doses of virus under the skin of rabbits and guinea-pigs, they do not become tuberculous; they lose weight toward the tenth day after inoculation, but after that gain weight and live indefinitely." Grancher et Martin, Courmont and Dor, Hericourt and Richet, should be mentioned also among those who made more or less successful attempts at immunization of small animals.

In 1892, Trudeau produced a marked degree of immunity in rabbits againts virulent human bacilli, by subcutaneous inoculations with living avian cultures. The test of immunity was made by injecting the human organism into the anterior chamber of the eye, where the process could be well observed. In most cases the infection was abortive, while in the control rabbits, the eye was destroyed.

In 1894, De Schweinitz reported the successful protection of guinea-pigs, a cow, and a calf, against virulent cultures by means of a human bacillus obtained from Trudeau, which had become attenuated by prolonged cultivation. Later (1897) Trudeau reported his success in protecting guinea-pigs with the same culture. The year 1892, however, brought out the most pronounced successes in immunization against tuberculosis yet obtained. In Germany, Behring published his results in protecting calves against virulent bovine cultures by intravenous injections of cultures of human origin.

A few months later Pearson and Gilliland, working in the laboratory of the State Live Stock Sanitary Board of Pennsylvania, gave the results of experiments which had been in progress for some time. By the use of a human sputum culture injected intravenously, they succeeded in protecting young cattle perfectly against highly virulent bovine cultures.

The results achieved in the recent past justify the strongest hope for the future. Those of us who are engaged in working out this problem realize perhaps better than any others the many difficulties in our path, and the many questions that remain yet to be answered. However, with the absolute demonstration that it is possible to immunize animals against the most virulent type of tubercle bacillus known, comes the conviction that the immunization of man must follow at no distant day. Whether this will be brought about as active immunization, through direct inoculations; or passive, through the serum of immunized animals, can not at present be answered. The trend of experiment at present is the production of a serum which will have a specific action on those already infected, and will give a passive immunity to healthy persons. Behring has proposed the immunization of children by feeding them on the milk of immunized cows. It has been shown by Maragliano that the milk of such animals has a certain amount of protective power, but it appears too feeble to be effective. Let us hope that means will be found whereby this power can be increased so that what is now a dream may become a blessed reality.

At the present time we have no specific against tuberculosis, but we have strong reasons to trust that the near future will place such a boon in our hands.—*Pennsylvania Medical Journal.*

